# Elevated Serum IgG Levels Positively Correlated with IL-27 May Indicate Poor Outcome in Patients with HBV-Related Acute-On-Chronic Liver Failure

**DOI:** 10.1155/2019/1538439

**Published:** 2019-05-06

**Authors:** Geng-lin Zhang, Qi-yi Zhao, Chan Xie, Liang Peng, Ting Zhang, Zhi-liang Gao

**Affiliations:** ^1^Department of Infectious Diseases, The Third Affiliated Hospital of Sun-Yat-sen University, Guangzhou 510630, China; ^2^Guangdong Provincial Key Laboratory of Liver Disease, The Third Affiliated Hospital of Sun-Yat-sen University, Guangzhou 510630, China; ^3^Department of Ultrasound, The Third Affiliated Hospital of Sun-Yat-sen University, Guangzhou 510630, China; ^4^Key Laboratory of Tropical Disease Control (Sun-Yat-sen University), Ministry of Education, Guangzhou 510630, China

## Abstract

**Background and Aims:**

Serum immunoglobulins are frequently increased in patients with chronic liver disease, but little is known about the role of serum immunoglobulins and their correlations with interleukin-27 (IL-27) in patients with HBV-related acute-on-chronic liver failure (HBV-ACLF). This study was aimed at determining the role of serum immunoglobulin (IgG, IgA, and IgM) levels and their associations with IL-27 in noncirrhotic patients with HBV-ACLF.

**Methods:**

Samples were assessed from thirty patients with HBV-ACLF, twenty-four chronic hepatitis B (CHB) subjects, and eighteen normal controls. Disease severity of HBV-ACLF was evaluated. Serum IL-27 levels were examined by enzyme-linked immunosorbent assay. Immunoglobulin levels were assessed using immunoturbidimetric assay. Correlations between immunoglobulin levels and IL-27 were analyzed. Receiver operating characteristic (ROC) curves were used to predict the 3-month mortality.

**Results:**

25 (83.3%) HBV-ACLF patients had elevated serum IgG levels (>1 ULN), 14 (46.7%) patients had elevated IgA, and 15 (50%) had raised IgM. IgG, IgA, and IgM levels were higher in HBV-ACLF patients than in CHB patients and normal controls. Moreover, IgG, IgA, and IgM levels were positively correlated with Tbil levels but negatively correlated with prothrombin time activity (PTA) levels. Additionally, IgG levels were significantly increased in nonsurviving patients than in surviving HBV-ACLF patients (*P* = 0.007) and positively correlated with MELD score (*r* = 0.401, *P* = 0.028). Also, IgG levels were positively correlated with IL-27 levels in HBV-ACLF patients (*r* = 0.398, *P* = 0.029). Furthermore, ROC curve showed that IgG levels could predict the 3-month mortality in HBV-ACLF patients (the area under the ROC curve: 0.752, *P* = 0.005).

**Conclusions:**

Our findings demonstrated that serum immunoglobulins were preferentially elevated in HBV-ACLF patients. IgG levels were positively correlated with IL-27 and may predict prognosis in HBV-ACLF patients.

## 1. Introduction

Acute-on-chronic liver failure (ACLF) is a dramatic clinical syndrome characterized by the sudden loss of hepatic cells leading to multiorgan failure in patients with preexisting chronic liver diseases [1]. The leading cause of ACLF in China is chronic HBV infection, while it is often a result of alcoholic cirrhosis in western countries [2]. An unclear pathogenesis of HBV-ACLF and the lack of effective treatment options result in an extremely high mortality rate. Substantial evidence indicates that immunity-mediated inflammation plays an essential role in HBV-ACLF. Particularly, different arms of the innate and adaptive immune cells make critical contributions to the development and progression of HBV-ACLF [3, 4].

Classically referred as core part of humoral immunity, immunoglobulins have been shown to play key roles in several types of liver diseases. Serum immunoglobulins are frequently elevated in chronic liver disease and cirrhotic patients [5, 6]. In addition, characteristic patterns of elevation in serum immunoglobulins are observed in specific liver diseases such as raised immunoglobulin G (IgG) in autoimmune hepatitis, raised immunoglobulin A (IgA) in alcoholic liver disease, and raised immunoglobulin M (IgM) in primary biliary cholangitis (PBC), which can be applied to aid diagnosis in clinical practice [7–10]. These pieces of evidence strongly link immunoglobulins with immune-mediated liver injury. However, less information has been available about the role of immunoglobulins in patients with HBV-ACLF.

Interleukin-27 (IL-27) is a relatively new cytokine that belongs to the IL-12 family. IL-27 is a heterodimeric cytokine composed of the Epstein-Barr virus-induced gene 3 (EBI3) and IL-27p28, which engages a receptor composed of gp130 and IL-27Ra that activates the JAK-STAT and MAPK signaling pathways [11]. IL-27 has both proinflammatory and anti-inflammatory properties that act on various types of cells depending on the context [11, 12]. Most studies mainly focus the effects of IL-27 on T cells. Also, IL-27 has been evidenced to regulate the expression of immunoglobulins by B cells. Several recent reports showed that IL-27 can induce the production of IgG1 by B cells and support antibody-driven autoimmune disease [13–15]. However, another report revealed that IL-27 can directly inhibit the growth of leukemic B cells [16]. To date, less is known about the correlations between IL-27 levels and the expression of immunoglobulins in patients with HBV-ACLF.

An important report enrolling patients with HBV-associated acute liver failure (HBV-ALF) clearly revealed that massive accumulation of plasma cells secreting IgG and IgM was found in the liver tissue [17]. In view of the similar pathogenic mechanisms between HBV-ALF and HBV-ACLF, we hypothesized that serum immunoglobulins could be elevated in patients with HBV-ACLF and that higher levels may indicate the unfavorable outcome. Therefore, in the present study, our aim was to determine the role of serum immunoglobulin levels (IgG, IgA, and IgM) and their correlations with IL-27 in noncirrhotic patients with HBV-ACLF.

## 2. Patients and Methods

### 2.1. Study Design and Patients

This study used clinical data and serum samples from our prospective study investigating the pathogenesis of HBV-ACLF patients. Briefly, thirty HBV-ACLF patients admitted to our department between June 2009 and May 2010 were enrolled. The inclusion criterion was based on the published works and clinical practice guideline. Adult noncirrhotic patients with HBV-ACLF who were willing to participate and consented to the study were enrolled based on previously described inclusion criteria [18, 19]. The exclusion criteria were the following: (1) evidence of other liver diseases including autoimmune liver diseases (autoimmune hepatitis and PBC), Wilson's disease, or cancer; (2) coinfection with other hepatitis virus or HIV virus; (3) treatment with artificial liver support or immunomodulatory drugs; (4) history of alcohol or drug abuse; and (5) records of renal, cardiovascular, pulmonary, or rheumatic diseases and pregnant women. Cirrhosis was clinically diagnosed when a small and nodular liver was found on imaging tests before enrollment [20]. Each patient was treated with supportive internal treatment. All HBV-ACLF patients were followed up at least 6 months. The clinical outcome was recorded as surviving or nonsurviving. Twenty-four chronic hepatitis B (CHB) patients and eighteen normal controls (NC) from our hospital during the same period were recruited as controls. CHB was diagnosed based on previously described criteria [18, 19]. Clinical assessment was performed at admission prior to therapy. Peripheral blood was collected at admission; serum was separated and stored at -80°C until being analyzed. The study was conducted in accordance with the Declaration of Helsinki, and the protocol was assessed and approved by our hospital's ethics committee. Written informed consent was obtained from each participant before the study.

### 2.2. Analysis of the Expression of IgG, IgA, and IgM

Human IgG, IgA, and IgM quantitation kits were purchased from Roche Diagnostics (Indianapolis, IN, USA). Serum concentrations of IgG, IgA, and IgM were analyzed using immunoturbidimetric assay through an autoanalyzer (TBA-30FR Toshiba, Tokyo, Japan) according to the manufacturer's guidelines. All values were compared to the normal ranges which were reported as 8-16 g/L for IgG, 0.7-3.3 g/L for IgA, and 0.5-2.2 g/L for IgM.

### 2.3. Analysis of IL-27 Levels by Enzyme-Linked Immunosorbent Assay

Serum IL-27 concentrations were quantified by sandwich enzyme-linked immunosorbent assay (ELISA) using commercial kits (BioLegend, San Diego, CA, USA) according to the manufacturer's protocol. Serum IL-27 levels were quantified by using standard samples with known cytokine concentrations provided by the manufacturer and expressed as pg/mL. The detection sensitivity was 11 pg/mL.

### 2.4. Virological Assessment and Liver Biochemical Assays

Serum HBV markers, including hepatitis B s antigen (HBsAg), hepatitis B e antigen (HBeAg), hepatitis B e antibody (HBeAb), and hepatitis B c antibody (HBcAb), were determined using the Elecsys system (Hoffmann-La Roche, Basel, Switzerland). HBV-DNA levels were quantitated by Real-Time Quantitative PCR using the ABI7300 (Applied Biosystems, Foster City, CA, USA). The limit of detection of HBV-DNA was 100 IU/mL. Liver biochemical assays were quantitated using an autoanalyzer (TBA-30FR Toshiba, Tokyo, Japan). Prothrombin time activity (PTA) was measured using an automatic hemostasis/thrombosis analyzer (STA Compact, Holliston, MA, USA).

### 2.5. Assessment of Complications and Disease Severity

Complete medical histories, physical examinations, and laboratory parameters were assessed for all HBV-ACLF patients. Complications (including spontaneous bacterial peritonitis, hepatic encephalopathy, hepatorenal syndrome, and upper gastrointestinal bleeding) were closely monitored and diagnosed based on our previously described standards [21]. Model for end-stage liver disease (MELD) score and MELD-Na score were used to assess disease severity and calculated as previously described [22]. If the sodium value was below 125 mmol/L, it was set to 125 mmol/L, and if the value was above 140 mmol/L, it was adjusted to 140 mmol/L. Moreover, the recently developed Chronic Liver Failure Consortium (CLIF-C) ACLF score for classification and prognostic assessment of ACLF patients was also applied to evaluate the disease severity [23]. CLIF-C ACLF score was calculated as follows: CLIF − C ACLF = 10 × [0.33 × CLIF − OFs + 0.04 × Age + 0.63 × ln (WBC count) − 2].

### 2.6. Statistical Analysis

Data were analyzed using SPSS version 20.0 software (IBM Corporation, Armonk, NY, USA) and expressed as frequencies, medians, and ranges or as means ± standard errors. Differences in variables were analyzed using ANOVA and Student's *t*-tests (for normally distributed data) or Kruskal-Wallis and Mann-Whitney *U* tests (for nonnormally distributed data). Categorical data were analyzed using the Chi-square test and Fisher's exact test. Correlation analysis was evaluated by the Pearson or Spearman test. Receiver operating characteristic (ROC) curves were used to predict the 3-month mortality. Comparisons of ROC curves were performed using the DeLong test. A two-sided *P* < 0.05 was considered statistically significant.

## 3. Results

### 3.1. Characteristics of Patients

The characteristics of HBV-ACLF, CHB, and normal controls are presented in Table 1. No significant differences existed among the three groups for the age (*P* = 0.073) or gender ratio (*P* = 0.718). Moreover, no statistically significant difference was found between the CHB and HBV-ACLF groups with respect to the presence of HBeAg (*P* = 0.061).

### 3.2. Serum Levels of IgG, IgA, and IgM Increased in HBV-ACLF Patients Independently of the Presence of HBeAg

**Figure 1:**
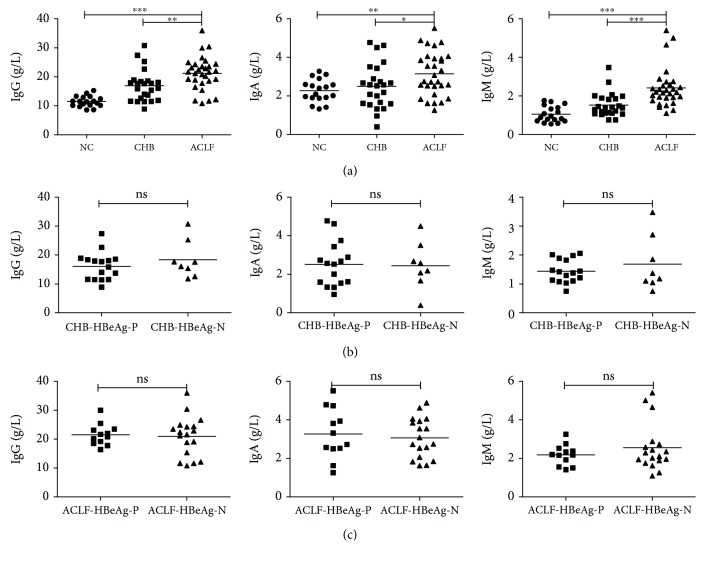
IgG, IgA, and IgM levels were significantly higher in HBV-ACLF patients independently of HBeAg presence. Pooled data indicated the levels of IgG, IgA, and IgM in each group, where the lines indicated the mean or median. (a) Serum IgG, IgA, and IgM levels were significantly higher in HBV-ACLF patients than in the CHB group and the NC group. No significant differences existed between patients with HBeAg positive and those with HBeAg negative, neither in the CHB group (b) nor in the HBV-ACLF group (c). ACLF: acute-on-chronic liver failure; CHB: chronic hepatitis B; NC: normal control; HBeAg-P: HBeAg-positive; HBeAg-N: HBeAg-negative; ∗*P* < 0.05; ∗∗*P* < 0.01; ∗∗∗*P* < 0.001; ns: not significant.

IgG, IgA, and IgM levels in serum samples from HBV-infected patients and uninfected controls were assessed using commercial kits. Overall, there were more patients with serum elevated immunoglobulins levels (defined as >1 time the upper limit of normal, >1 ULN) in the HBV-ACLF group when compared with the CHB group for elevated IgG levels (83.3% vs. 54.2%, *P* = 0.034), for elevated IgA levels (46.7% vs. 25%, *P* = 0.156), and for elevated IgM levels (50% vs. 8.3%, *P* = 0.001). Moreover, serum IgG levels were significantly increased in patients with HBV-ACLF (mean 21.21 g/L) when compared with CHB subjects (median 16.83 g/L, *P* = 0.002) and normal controls (mean 11.44 g/L, *P* < 0.001; Figure 1(a)). Also, serum IgA levels were significantly increased in patients with HBV-ACLF (mean 3.15 g/L) than in CHB subjects (mean 2.50 g/L, *P* = 0.042) and normal controls (mean 2.27 g/L, *P* = 0.001; Figure 1(a)). Moreover, serum IgM levels were significantly increased in patients with HBV-ACLF (median 2.19 g/L) when compared with CHB subjects (median 1.38 g/L, *P* < 0.001) and normal controls (median 0.92 g/L, *P* < 0.001; Figure 1(a)). We then determined the correlations between HBeAg presence and immunoglobulin levels. In the CHB group, no significant differences existed in IgG, IgA, or IgM levels between HBeAg-positive (*n* = 16) and HBeAg-negative patients (*n* = 8) (*P* = 0.337, *P* = 0.890, and *P* = 0.503, resp.; Figure 1(b)). Also, no significant differences existed in IgG, IgA, or IgM levels between HBeAg-positive HBV-ACLF patients (*n* = 12) and patients with HBeAg-negative HBV-ACLF (*n* = 18) (*P* = 0.776, *P* = 0.634, and *P* = 0.755, resp.; Figure 1(c)).

### 3.3. Increased Serum IgG, IgA, and IgM Levels Were Closely Associated with Liver Injury in HBV-Infected Patients

**Figure 2:**
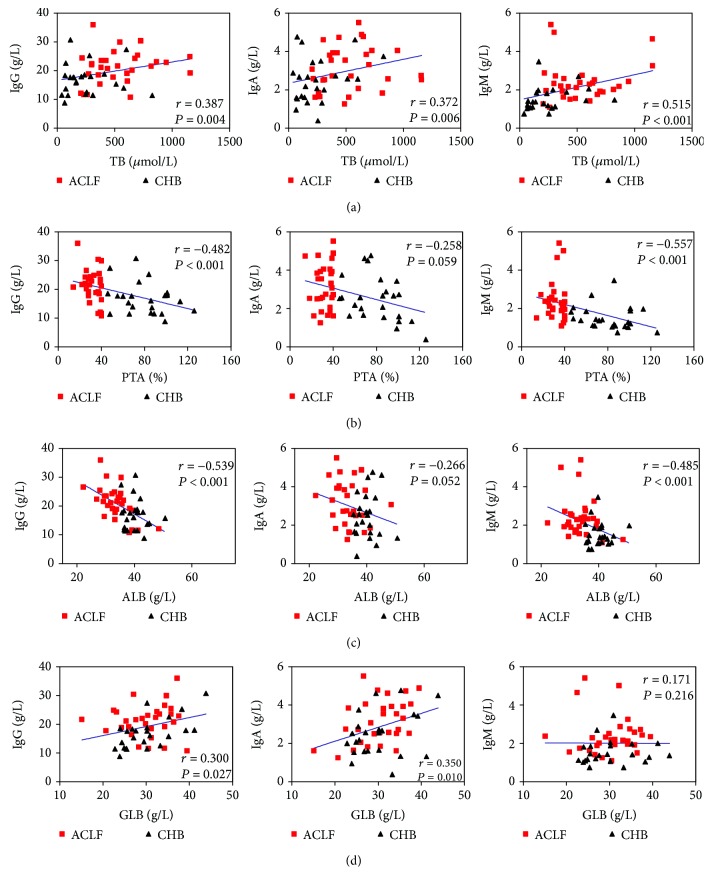
Elevated serum IgG, IgA, and IgM may contribute to liver injury. Serum IgG, IgA, and IgM levels were positively correlated with serum total bilirubin (TB) levels (a) but negatively associated with plasma prothrombin time activity (PTA) levels (b) and serum albumin (ALB) levels (c). In addition, positive correlations were found between immunoglobulin (IgG and IgA) and serum globulin (GLB) levels (d) in HBV-infected patients. Solid line: linear growth trend; *r*: correlation coefficient. *P* values are shown.

We subsequently analyzed the correlations between IgG, IgA, and IgM levels and serum total bilirubin (Tbil), plasma PTA, serum albumin, serum globulin, serum ALT, serum AST levels, and serum HBV-DNA loads in CHB and HBV-ACLF patients. Interestingly, serum Tbil levels were positively correlated with IgG, IgA, and IgM levels (*r* = 0.387, *P* = 0.004; *r* = 0.372, *P* = 0.006; and *r* = 0.515, *P* < 0.001, resp.; Figure 2(a)), while plasma PTA levels were negatively correlated with IgG, IgA, and IgM levels (*r* = −0.482, *P* < 0.001; *r* = −0.258, *P* = 0.059; and *r* = −0.557, *P* < 0.001, resp.; Figure 2(b)) in these HBV-infected subjects. In addition, serum albumin levels were negatively correlated with IgG, IgA, and IgM levels (*r* = −0.539, *P* < 0.001; *r* = −0.266, *P* = 0.052; and *r* = −0.485, *P* < 0.001, resp.; Figure 2(c)). Serum globulin levels were positively correlated with IgG and IgA levels (*r* = 0.300, *P* = 0.027 and *r* = 0.350, *P* = 0.010; Figure 2(d)). However, no significant correlations existed between IgG, IgA, or IgM levels and ALT levels, AST levels, or HBV-DNA levels in these HBV-infected patients (all *P* > 0.05). These findings suggest that increased serum IgG, IgA, and IgM levels were closely associated with liver injury in HBV-infected patients.

### 3.4. IgG Levels Were Closely Associated with Clinical Outcome in HBV-ACLF Patients

**Figure 3:**
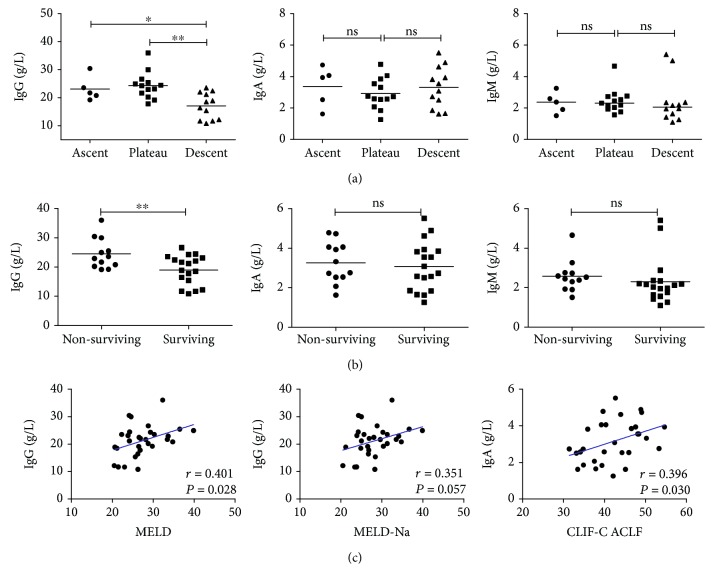
IgG levels were closely associated with disease severity in HBV-ACLF patients. (a) IgG levels were significantly lower in HBV-ACLF patients at the descent stage than patients at the ascent stage (*P* = 0.022) and patients at the plateau stage (*P* = 0.001). However, no significant differences existed in IgA or IgM levels among HBV-ACLF patients at different stages. (b) Serum IgG levels were significantly higher in nonsurviving HBV-ACLF patients than in surviving patients (*P* = 0.007). Also, we noticed that serum IgM levels were slightly higher in nonsurviving HBV-ACLF patients than in surviving patients (*P* = 0.079). (c) Positive correlations were found between IgG levels and MELD score, between IgA levels and CLIF-C ACLF score. And a positive correlation trend was found between IgG levels and MELD-Na score. ∗*P* < 0.05; ∗∗*P* < 0.01; ns: not significant; solid line: linear growth trend; *r*: correlation coefficient.

We stratified patients into three stages according to the natural history of HBV-ACLF [24, 25], the ascent stage (*n* = 5), the plateau stage (*n* = 13), and the descent stage (*n* = 12). Interestingly, IgG levels were significantly lower in patients at the descent stage (17.04 ± 1.35 g/L) than in patients at the ascent stage (23.11 ± 1.96 g/L, *P* = 0.022) and in patients at the plateau stage (24.33 ± 1.32 g/L, *P* = 0.001). However, no significant differences existed in IgA or IgM levels among HBV-ACLF patients at different stages (Figure 3(a)). During the follow-up period, eighteen HBV-ACLF patients survived, while twelve patients died. Then, we examined the correlations between clinical outcome and expression of IgG, IgA, and IgM. Serum IgG levels were significantly higher in nonsurviving HBV-ACLF patients (24.51 ± 1.51 g/L, *n* = 12) than in surviving patients (19.02 ± 1.18 g/L, *n* = 18, *P* = 0.007; Figure 3(b)). Also, we noticed that serum IgM levels were slightly higher in nonsurviving HBV-ACLF patients (median 2.48 g/L) than in surviving HBV-ACLF patients (median 2.07 g/L, *P* = 0.079; Figure 3(b)). However, there was no significant difference in IgA levels between nonsurviving HBV-ACLF patients (3.26 ± 0.30 g/L) and those surviving (3.08 ± 0.29 g/L, *P* = 0.674; Figure 3(b)). MELD score, MELD-Na score, and CLIF-C ACLF score are widely applied to assess disease severity in HBV-ACLF. In this study, these parameters were calculated as described at admission. The results were 27.37 ± 0.89, 28.24 ± 0.84, and 42.20 ± 1.15 for MELD score, MELD-Na score, and CLIF-C ACLF score, respectively. Next, the correlations between immunoglobulin levels (IgG, IgA, and IgM) and these parameters were determined by Pearson analysis. Interestingly, positive correlations were found between IgG levels and MELD score (*r* = 0.401, *P* = 0.028), between IgA levels and CLIF-C ACLF score (*r* = 0.396, *P* = 0.030; Figure 3(c)). And a positive correlation trend was found between IgG levels and MELD-Na score (*r* = 0.351, *P* = 0.057; Figure 3(c)). However, no significant correlations existed between IgM levels and these scores in HBV-ACLF patients (all *P* > 0.05).

### 3.5. IL-27 Levels Were Positively Correlated with Immunoglobulins in HBV-Infected Patients

**Figure 4:**
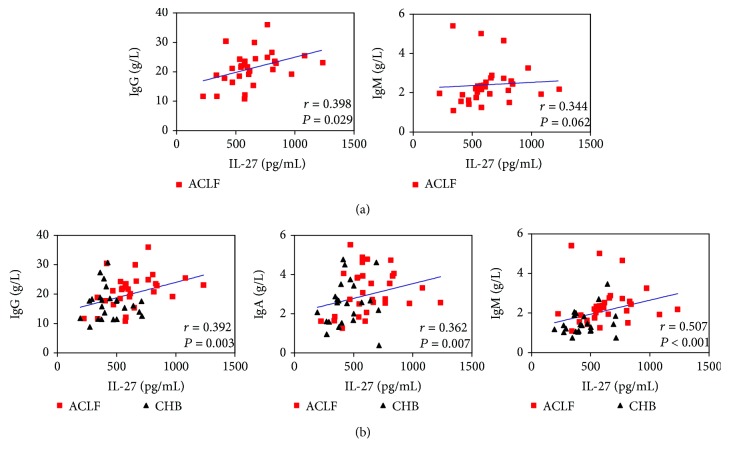
IL-27 levels were positively correlated with the expression of immunoglobulins. (a) IgG levels were found to be positively correlated with IL-27 levels; and a positive correlation trend was found between IgM levels and IL-27 levels in HBV-ACLF patients. (b) Positive correlations were found between IL-27 levels and serum immunoglobulin levels (IgG, IgA, and IgM) in HBV-infected patients. Solid line: linear growth trend; *r*: correlation coefficient. *P* values are shown.

IL-27 has been evidenced to regulate the production of immunoglobulin by B cells. In this study, serum IL-27 levels were significantly higher in HBV-ACLF patients (634.58 ± 40.21 pg/mL) than in the CHB group (441.25 ± 28.87 pg/mL, *P* < 0.001) and the NC group (277.14 ± 23.96 pg/mL, *P* < 0.001). Next, the correlations between IL-27 levels and immunoglobulin levels were examined by Spearman or Pearson analysis. Interestingly, IgG levels were positively correlated with IL-27 levels (*r* = 0.398, *P* = 0.029), and a positive correlation trend was found between IgM levels and IL-27 levels (*r* = 0.344, *P* = 0.062; Figure 4(a)) in HBV-ACLF patients. Furthermore, positive correlations were found between IL-27 levels and IgG levels (*r* = 0.392, *P* = 0.003), IgA levels (*r* = 0.362, *P* = 0.007), and IgM levels (*r* = 0.507, *P* < 0.001; Figure 4(b)) in HBV-infected patients. These results indicate that IL-27 may induce the production of immunoglobulins in HBV-infected patients.

### 3.6. Higher IgG Levels May Predict Poor Prognosis in HBV-ACLF Patients

**Figure 5:**
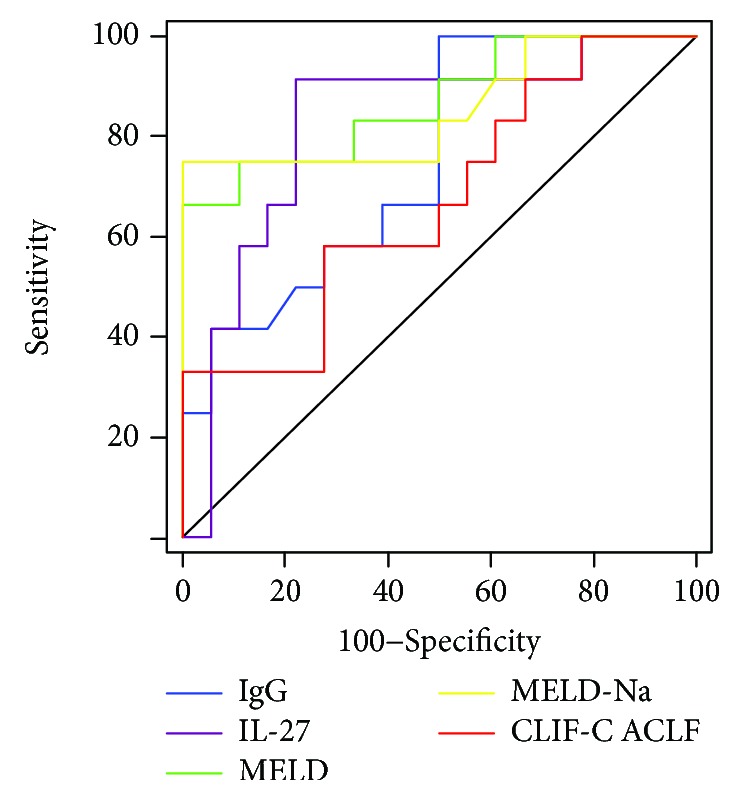
Accuracy of IgG levels was compared to other parameters in predicting the 3-month mortality of HBV-ACLF patients. The area under the ROC curve (AUC) for IgG levels was 0.752 (95% confidence interval: 0.562-0.891, *P* = 0.005). Interestingly, no significant differences existed between AUC values obtained using IgG levels and those obtained with IL-27 levels (0.824, *P* = 0.531), MELD score (0.870, *P* = 0.320), MELD-Na score (0.854, *P* = 0.433), or CLIF-C ACLF score (0.671, *P* = 0.536) in HBV-ACLF patients.

The 3-month mortality rate is a widely accepted indicator for the long-term prognosis of HBV-ACLF patients [23, 24]. In the current study, twelve HBV-ACLF patients died in the first three months, while eighteen patients survived during the follow-up period. Thus, the 3-month mortality rate was 40%. Finally, IgG levels were assessed to predict the 3-month mortality of HBV-ACLF patients and compared with IL-27 levels and disease severity scores (MELD, MELD-Na, and CLIF-C ACLF) by ROC curves (Figure 5, Table 2). The area under the ROC curve (AUC) for IgG levels was 0.752 (95% confidence interval: 0.562-0.891, *P* = 0.005). Interestingly, no significant differences existed between AUC values obtained using IgG levels and those obtained with IL-27 levels (0.824, *P* = 0.531), MELD score (0.870, *P* = 0.320), MELD-Na score (0.854, *P* = 0.433), or CLIF-C ACLF score (0.671, *P* = 0.536), indicating that IgG levels may have prognostic value equivalent to these parameters.

## 4. Discussion

To date, less information has been available on the role of serum immunoglobulins and their correlations with IL-27 in patients with HBV-ACLF. This study shows that there were more patients with elevated immunoglobulin levels in the HBV-ACLF group than in the CHB group; and immunoglobulin (IgG, IgA, and IgM) levels were positively linked with liver injury. In addition, IgG levels were positively associated with MELD score (*r* = 0.401, *P* = 0.028) and may predict the 3-month mortality using ROC curves (AUC = 0.752, *P* = 0.005) in patients with HBV-ACLF. Furthermore, this study demonstrates that IgG levels were positively correlated with the expression of IL-27 in patients with HBV-ACLF (*r* = 0.398, *P* = 0.029), indicating that IL-27 may participate in the production of IgG.

Previous studies have shown that specific elevations of serum immunoglobulin are found in several types of liver disease, such as autoimmune hepatitis (elevated IgG), PBC (elevated IgM), and alcoholic liver disease and nonalcoholic fatty liver disease (elevated IgA) [7–10, 26]. However, little is known about how serum immunoglobulin levels are altered in patients with HBV-ACLF. In the present study, 83.3% HBV-ACLF patients had an elevated IgG (>1 ULN), whereas 46.7% patients had an elevated IgA (>1 ULN) and 50% patients had an elevated IgM (>1 ULN). These data indicate a polyclonal increase of immunoglobulins, which may be a characteristic of patients with HBV-ACLF. Therefore, when investigating patients with suspected liver disease, the finding of a polyclonal increase of immunoglobulins should prompt clinicians to consider HBV-ACLF as a diagnosis when there is no evidence of autoimmune liver diseases and no history of excessive alcohol consumption.

T lymphocytes have been evidenced to play an essential role in the pathogenesis of HBV-ACLF [27]. However, studies on B lymphocytes are limited. Also, the role of the immunoglobulins in HBV-ACLF patients is poorly understood. A recent study using liver tissue showed that B lymphocyte-mediated responses were highly active during the immune tolerance and immune active phases of patients with chronic hepatitis B [28]. Moreover, an overwhelming B cell response was seen, and massive production of immunoglobulin by plasma cells was found to be deposited in the liver parenchyma in patients with HBV-associated acute liver failure [17]. These studies led us to hypothesize that immunoglobulins produced by B cells may contribute to liver injury in HBV-ACLF. There are several lines of evidence to support this notion. Firstly, we demonstrate that HBV-ACLF patients had higher serum IgG, IgA, and IgM levels compared to CHB patients and normal controls. In addition, serum IgG, IgA, and IgM levels in HBV-infected patients were positively associated with serum Tbil levels but negatively correlated with PTA levels and ALB levels, which are often served as markers of liver injury [29]. Moreover, IgG levels were positively correlated with MELD score, which is an important indicator of disease severity in HBV-ACLF. And, IgG levels were significantly higher in nonsurviving HBV-ACLF patients than in surviving patients. Furthermore, the ROC curve analysis showed that IgG levels accurately predicted the 3-month mortality in patients with HBV-ACLF (AUC = 0.752, *P* = 0.005). These results suggest that IgG plays an important role in the pathogenesis of HBV-ACLF, and higher levels of IgG may predict poor prognosis.

Cytokines serving as mediators of immune responses play an essential role in the mechanism of immune-mediated diseases. IL-27, a heterodimeric cytokine comprising the IL-27p28 and EBI3 subunits, is a member of the IL-6/IL-12 family of cytokines and can exert proinflammatory and anti-inflammatory effects during immune responses [11, 12]. B cells express the complete IL-27R, which can be detected on human naïve B cells, memory B cells, and resting plasma cells [30]. More importantly, IL-27 has been evidenced to support antibody-driven autoimmune diseases through both direct and indirect effects on B cells [14, 15, 31], whereas another report revealed that IL-27 can directly inhibit the growth of leukemic B cells [16]. To date, less is known about the role of IL-27 on the expression of immunoglobulins in patients with HBV-ACLF. To our knowledge, the current study is the first one to examine the correlations between IL-27 expression and immunoglobulins in HBV-ACLF. In accordance with our previous report [18], IL-27 levels were found to be significantly increased in HBV-ACLF patients than in the CHB group and the NC group in the present study. Next, we demonstrated that immunoglobulins were positively correlated with serum IL-27 levels in HBV-infected patients (Figure 4). More importantly, IgG levels were positively associated with IL-27 levels (*r* = 0.398, *P* = 0.029), and a positive trend between IgM levels and IL-27 levels (*r* = 0.344, *P* = 0.062) was found in patients with HBV-ACLF. Collectively, these data indicate that the preferential elevated immunoglobulins were closely linked with IL-27 levels. IL-27 may participate in the production of IgG in HBV-ACLF, but the exact mechanism should be intensely explored and further studies are required.

This study has several limitations. The intrahepatic expressions of immunoglobulins were not determined in patients with HBV-ACLF due to the poor coagulation, which is considered to be a contraindication for liver biopsy. Also, further studies are necessary to investigate the direct effects of IL-27 on the production of immunoglobulins by B lymphocytes in patients with HBV-ACLF. Moreover, this study was retrospective with a limited sample size; consequently, the results should be further validated in larger studies.

In summary, our findings demonstrate that a polyclonal increase of immunoglobulins existed and was closely linked with liver injury in patients with HBV-ACLF. Furthermore, immunoglobulin levels were positively correlated with IL-27 levels, suggesting that IL-27 may help induce the expression of immunoglobulins. In addition, higher IgG levels could predict poor prognosis in HBV-ACLF. Therefore, along with other clinical features and biochemical results, assessment of serum IgG levels could help physicians identify higher-risk HBV-ACLF patients earlier.

## Figures and Tables

**Table 1:** Clinical characteristics of the participants enrolled in the study.

Groups	NC (*n* = 18)	CHB (*n* = 24)	HBV-ACLF (*n* = 30)
Gender (male)	17	24	29
Age (years)	36.33 ± 2.84	36.92 ± 1.50	42.97 ± 2.44
ALT (U/L)	22.67 ± 2.18	186.5 (34-2424)	187.5 (27-2879)
AST (U/L)	23.39 ± 1.75	211.5 (56-2646)	163 (66-1859)
Tbil (*μ*mol/L)	N.D.	186.9 (39.1-826.2)	489.6 (204.5-1157.0)
PTA (%)	N.D.	85 (46-126)	34 (14-40)
Albumin (g/L)	N.D.	40.2 (35.4-50.7)	33.53 ± 0.92
Globulin (g/L)	N.D.	29.9 (22.8-43.9)	30.22 ± 1.03
HBV-DNA (log_10_IU/mL)	N.D.	5.33 ± 0.24	4.59 ± 0.25
Complication	0	0	22
SBP	0	0	19
Hepatic encephalopathy	0	0	11
Hepatorenal syndrome	0	0	2
UGB	0	0	1
MELD score	N.D.	N.D.	27.37 ± 0.89
MELD-Na score	N.D.	N.D.	28.24 ± 0.84
CLIF-C ACLF score	N.D.	N.D.	42.20 ± 1.15
IgG (g/L)	11.44 ± 0.44	16.83 (8.86-30.76)	21.21 ± 1.04
Elevated IgG (>1 ULN)	0	13	25
IgA (g/L)	2.27 ± 0.14	2.50 ± 0.24	3.15 ± 0.21
Elevated IgA (>1 ULN)	0	6	14
IgM (g/L)	0.92 (0.54-1.75)	1.38 (0.75-3.47)	2.19 (1.09-5.41)
Elevated IgM (>1 ULN)	0	2	15
HBsAg positive	0	24	30
HBsAb positive	18	0	0
HbeAg positive	0	16	12

Data are shown as means ± standard errors or medians and ranges. ACLF: acute-on-chronic liver failure; CHB: chronic hepatitis B; NC: normal control; ALT: alanine aminotransferase; AST: aspartate aminotransferase; Tbil: total bilirubin; PTA: prothrombin time activity; SBP: spontaneous bacterial peritonitis; UGB: upper gastrointestinal bleeding; MELD: model for end-stage liver disease; CLIF-C: Chronic Liver Failure Consortium; IgG: immunoglobulin G; IgA: immunoglobulin A; IgM: immunoglobulin M; ULN: upper limit of normal; N.D: not determined.

**Table 2:** Comparisons of the area under the ROC curves (AUCs) estimated for each parameter.

Parameters	IL-27 (g/L)	IgG (g/L)	MELD	MELD-Na	CLIF-C ACLF
AUC	0.824 (0.642-0.938)	0.752 (0.562-0.891)	0.870 (0.697-0.964)	0.854 (0.678-0.956)	0.671 (0.477-0.831)
*P* value vs. IgG	0.531	-	0.320	0.433	0.536

Data in parentheses are 95% confidence interval. IgG: immunoglobulin G; MELD: model for end-stage liver disease; CLIF-C: Chronic Liver Failure Consortium; AUC: area under the ROC curve.

## Data Availability

The data used or analyzed during the current study are available from the first author (Geng-lin Zhang) on reasonable request.

## Abbreviations

ACLF: Acute-on-chronic liver failure
CHB: Chronic hepatitis B
NC: Normal control
HBV: Hepatitis B virus
Tbil: Total bilirubin
PTA: Prothrombin time activity
MELD: Model for end-stage liver disease
CLIF-C: Chronic Liver Failure Consortium
IgG: Immunoglobulin G
IgA: Immunoglobulin A
IgM: Immunoglobulin M
IL-27: Interleukin-27
ULN: Upper limit of normal.
